# Automatic multi-IMU-based deep learning evaluation of intensity during static standing balance training exercises

**DOI:** 10.1186/s12984-025-01760-3

**Published:** 2025-11-27

**Authors:** Safa Jabri, Jeremiah Hauth, Christopher DiCesare, Wendy Carender, Lauro Ojeda, Jenna Wiens, Leia Stirling, Xun Huan, Kathleen H. Sienko

**Affiliations:** 1 University of Michigan–Ann Arbor, Ann Arbor, USA; 2 Michigan Medicine, Ann Arbor, USA

**Keywords:** Balance, Balance rehabilitation, Physical therapy, Wearable sensors, IMU, Machine learning, Standing balance.

## Abstract

**Background:**

Effective balance rehabilitation requires training at an appropriate level of exercise intensity given an individual’s needs and abilities. Typically balance intensity is assessed through in-clinic visual observation by physical therapists (PTs), which limits the ability to monitor and progress intensity during home-based components of training programs. The goal of this study was to train and evaluate machine learning models for estimating physical therapists’ perceived balance exercise intensity using data from full-body wearable sensors to support the development of home-based training exercise dosage monitoring.

**Methods:**

Balance exercise participants (*n* = 47) participated in a single-day balance training session where they were filmed performing static standing exercises at various levels of intensity. Kinematic data from 13 full-body wearable inertial measurement units (IMUs) and self-ratings of balance intensity were also collected. An additional cohort of PT participants (*n* = 42) was recruited to watch the videos of the balance exercise participants and provide ratings of balance intensity. The mean PT rating for each video was used as a ground truth (GT) label of balance intensity. We trained and evaluated Convolutional Neural Networks (CNN)-based models to predict balance intensity based on performance as captured through the IMUs. Model performance was evaluated by calculating the root-mean-square error (RMSE) of predications. A sensitivity analysis was also performed to assess the effect of the number of IMUs used on model performance.

**Results:**

Models trained on orientation derived from all 13 IMUs achieved good predictive performance as indicated by a RMSE of 0.66 [0.62, 0.69], which was within the threshold defined by typical inter-rater variabilities between PTs (RMSE of 0.74 [0.72, 0.76]). Sensitivity analysis indicated that model performance stabilized at four sensors with the best performance corresponding to sensors placed on both thighs and the lower and upper back.

**Conclusions:**

Findings from this study indicated that balance intensity assessment can be achieved through wearable sensors and a CNN model, which could support the supervision and effectiveness of home-based balance rehabilitation.

## Background

Balance deficits due to aging, injury, or neurosensory conditions affect the ability to maintain posture and increase the risk of falls. Balance training programs guided by physical therapists (PTs) support the recovery and maintenance of balance ability as they train individuals to utilize, reweigh, and integrate available sensory information (i.e., visual, vestibular, and neuromuscular inputs) under various balance conditions [1].

Effective balance training relies on appropriate dosage. In balance training, dosage is determined using the FITT framework [2], which stands for frequency (how often training occurs), intensity (the level of challenge in training), time (the duration of the balance exercise session), and type (the specific type of balance exercise performed). While frequency, time, and type of exercise are relatively simple to measure, assessing intensity in the context of balance training is more complex. The intensity of a balance exercise depends on the numerous neurosensory and motor demands involved in the exercise and is person-specific, often varying significantly due to the underlying conditions that contribute to balance deficits, physical limitations, and contextual factors [3]. Best practice recommendations stated by Sherrington et al. [4] advise that moderate to highly challenging balance exercises be performed at least 2 h per week to enhance balance and reduce the risk of falling in older adults. Similarly, Reinthal [5] stated that “exercise must be performed near an individual’s capacity” to induce a training effect, highlighting the importance of training at an appropriate level of intensity.

Customizing balance exercise programs to provide adequate levels of balance intensity supports the effectiveness of balance rehabilitation [1]. In clinical settings, PTs typically review medical histories and observe patients’ balance performance by evaluating biomarkers of balance intensity (e.g., body sway and compensatory reactions at the ankles, hips, trunk and upper extremities) [11] to tailor rehabilitation programs accordingly, ensuring that the level of challenge aligns with each individual’s needs and capabilities. A randomized controlled clinical trial by Shepard et al. [6] found that a customized vestibular rehabilitation therapy (VRT) program tailored to individual needs resulted in a higher likelihood of successful resolution of vestibular symptoms than a generic program. Multiple studies have since highlighted the value of customizing VRT programs for individuals with vestibular deficits [7–9]. Similarly, the customization of training programs was reported to enhance their effectiveness in older adults [10, 11]. However, due to the lack of a direct or standardized measure of balance intensity [3], program customization currently relies on in-person observational assessments by PTs.

Despite its effectiveness compared to unsupervised training, access to in-person physical therapy with PTs may be limited by patient load, insurance reimbursement policies, and travel to clinics [13, 14]. Typically, balance rehabilitation programs for individuals at fall risk rely on home-based unsupervised training to achieve the frequency of exercise required, along with interspersed in-clinic assessments to adjust and progress exercise plans. A systematic review and meta-analysis performed by Lacroix et al. [12] suggested that supervised balance training programs improved outcome measures in healthy older adults to a greater degree relative to unsupervised training (i.e., performed at home). In the absence of supervision by PTs during home-based training, intensity is typically not assessed, thus limiting the ability to monitor the effectiveness of a given training session. Developing alternative approaches to measure balance intensity in training programs without relying on PTs presents an opportunity to support effective rehabilitation, especially in the context of the rising interest in telerehabilitation as a means to bridge the gap in access posed by structural and societal limitations [13, 15], and address patients’ needs in the context of public health incidents such as the COVID-19 pandemic [16–18].

Sensor modalities that capture human movements such as motion capture (MoCAP) [19, 20], force plates [21–23], and inertial measurement units (IMUs) [24–30] have been widely studied as a means to evaluate balance performance. While MoCAP and force plate systems have typically been constrained to research settings due to the need for specialized equipment, IMUs have been widely incorporated in commercial products [31–33] that could support balance intensity quantification in home-based training. Since balance intensity is conceptualized as a product of exercise demands and the individual’s capacity assessed through observations of performance [5, 11], IMU-based kinematic measurements of balance performance may provide a reliable measure of balance intensity in real-world settings. In a recent study, Ferris et al. [34] examined the differences among PT ratings of balance intensity (1–5 scale), balance exercise participant self-ratings of perceived intensity (1–5 scale), and IMU-based kinematic measures of postural sway for static standing balance exercises. Study results revealed that balance intensity ratings derived from linear statistical models applied to kinematic data obtained from an IMU positioned on the lower back displayed a significant correlation with PT ratings of balance intensity, as evidenced by a Spearman correlation coefficient of *r* = 0.698, 95% confidence interval (CI) [0.675, 0.721]. However, it is worth noting that the classification accuracy of these models was found to be moderately low, falling within the range of 43.0% to 52.4%. In addition, balance exercise participants overestimated their performance, resulting in better self-ratings of balance intensity relative to PT ratings. While this study already observed a strong correlation between PT ratings of balance intensities and ratings predicted from IMU-based kinematic measurements using a linear model, the IMU-based rating predictions may be further improved through more complex machine learning (ML) approaches.

ML-based approaches to assessing balance intensity or evaluating balance exercise performance have been explored in a few recent studies. Bao et al. [35] and Kamran et al. [36] developed ML models based on kinematic data collected from wearable IMUs placed on the lower back during static standing balance exercises. Both studies showcased the potential of IMU-based models in automatic assessment of balance intensity to inform the dosage and progression of training. Such automated balance intensity assessment systems could support home-based components of balance training programs by providing feedback on training intensity to ensure effective and safe levels of challenge. However, by only relying on a single IMU placed on the lower back, the models presented by Bao et al. and Kamran et al. are only able to capture performance through trunk sway without considering other reported biomarkers of balance intensity [11], such as known compensatory strategies to balance perturbations at the ankles, knees, hips, and upper extremities. Including additional IMUs placed on various body segments may enable better predictive performance, by allowing the models to learn additional relevant information about the compensatory reactions a balance exerciser may engage in beyond trunk sway during a challenging balance exercise.

In a recent study, Tsakanikas et al. [39] collected data from 19 older adults at fall risk and developed models to score performance on a variety of balance training exercises, including two static standing exercises, using a network of synchronized sensors that included IMUs placed on the head and trunk, pressure insoles, and a depth camera. Engineered features extracted from these sensor modalities were used to predict PT ratings on a 0–3 scale with classification accuracies ranging from 85% to 92% on the standing exercises. These findings highlighted the feasibility of sensor-based performance evaluation using a collection of wearable devices (IMUs and insoles) and environmental monitoring (depth cameras) modalities. However, by relying on multiple modalities of data collection including cameras, their proposed approach may be affected by field-of-view restrictions associated with camera-based systems and pose implementation challenges in home-based applications.

While prior studies highlighted the potential for wearable IMUs and ML approaches to automatically evaluate balance intensity in the context of static standing exercises, none of these studies investigated the use of only full-body wearable sensor systems. The goal of this study was to train and evaluate ML models for estimating PTs’ perceived balance exercise intensities using data from full-body wearable sensors. To achieve this goal, we (1) developed ML models to evaluate balance intensity for prescribed balance rehabilitation exercises using a full body set of wearable IMUs and (2) identified sensor locations that best enabled these evaluations. We trained CNN-based deep learning models on a dataset of kinematic data from 13 IMU placements collected during balance exercises of varying intensity to predict PT ratings on a 1–5 balance intensity scale. The number of IMUs used and their placements were then analyzed to identify a minimal subset of informative sensor placements. The developed models were evaluated on held-out test data to assess their generalizability and their performance was compared to existing benchmarks. The models presented in this study represent a step towards the development of data-driven IMU-based tools for the assessment of balance intensity and exercise dosage to support balance training.

## Methods

### Overview

The purpose of this study was to develop and evaluate IMU-based data-driven models for the automatic evaluation of balance exercise intensity during static standing exercises. To accomplish this goal, a study protocol was developed whereby two human participant cohorts were recruited: (1) balance exercise participants who participated in a balance training session to capture the kinematic data used as inputs in the model development, and (2) balance evaluation participants consisting of PTs who observed the former cohort via video recordings and provided balance intensity ratings used as labels in model development. Based on this dataset, CNN-based models were trained to automatically predict PT ratings for a given balance exerciser, exercise, and orientation angles derived from wearable IMUs. Additional analyses were performed to assess the effect of the number of IMUs and their placement on model performance.

Data collection was performed under COVID-19 prevention protocols that required balance exercise participants to wear face masks during the training session. The study protocol was reviewed and approved by the University of Michigan Institutional Review Board (HUM00086479).

## Participants

### Balance exercise participants

Forty-seven participants (51 ± 18y; 30 female, 17 male) were recruited to participate in a single balance exercise session comprising static standing exercises from typical balance training sessions. The balance exercise participants were included if they reported being in good general health or if they had been diagnosed with a neuropathology, including a vestibular deficit, peripheral neuropathy, traumatic brain injury, multiple sclerosis (MS), and/or Parkinson’s disease (early to mid-stage). Inclusion criteria also included being able to demonstrate intact cognition, read and understand English, stand for up to 10 min at a time, and walk a city block without using an assistive device (e.g., cane, walker). Participants were excluded if they had a BMI >35 kg/m^2^ to minimize the confounding effects of atypical sway associated with higher BMI on balance measurements [40], had severe uncorrected vision or hearing loss impairing their ability to complete the balance exercises, or sustained a fall that resulted in hospitalization or serious injury in the past 12 months. We included participants without any diagnosed balance-related disorders as well as individuals with self-reported diagnosed balance disorders to build a dataset of balance performance at variable levels of balance ability (Table 1).

**Table 1:** Overview of balance exercise participants’ demographics

Participant ID #	Age (years)	Sex (F/M)	Height (cm)	Weight (kg)	Self-selected gait speed (m/s)	MiniBest (/28)	TUG (s)	TUG Dual Task (s)	Condition (self-report)
1	52	F	160	63.6	1.2	24	8.6	9.7	MS
2	60	F	173	93	1.4	22	9.2	10.4	MS
3	44	F	176	94.1	0.9	21	14.2	13.6	MS
4	25	M	176	67	1.4	27	7.5	8.5	–
5	27	F	164	57.05	1.2	27	8.2	9.3	–
6	25	M	158	53.35	1.2	28	8.4	8.6	–
7	23	F	173	62.55	1.5	28	7.1	8.0	–
8	25	F	163	59.6	1.1	27	6.5	5.9	–
9	47	F	162	75.5	1.1	25	9.1	11.3	–
10	21	F	155	60.55	1.5	28	6.6	6.9	–
11	19	F	171	62.6	1.2	27	7.3	10.9	–
12	49	M	173	76.65	1.7	26	7.1	7.3	–
13	25	F	169	54.55	1.5	28	7.5	8.0	–
14	54	F	178	86.6	1.5	25	8.5	9.0	–
15	46	M	180	98.2	1.6	27	7.2	8.5	–
16	60	F	165	67.15	1.1	24	7.8	9.0	–
17	31	F	168	56.95	1.6	27	6.6	8.1	–
18	62	F	170	74	1.3	27	8.3	10.3	–
19	66	M	180	94.6	1.6	26	8.8	9.3	–
20	41	F	162	58.2	1.0	27	8.0	8.8	–
21	20	F	170	69.15	1.3	27	7.8	9.4	–
22	65	F	159	52.35	1.3	27	8.6	9.3	–
23	67	F	152	54.9	0.9	24	8.0	12.3	–
24	69	M	173	88.6	1.4	25	8.1	9.7	Parkinson’s Disease
25	74	M	175	74.9	1.3	24	9.0	9.3	–
26	66	F	170	51.95	1.1	23	9.0	9.1	–
27	68	M	182	86.65	1.3	26	9.6	10.6	–
28	67	F	184	67.8	1.4	26	8.6	9.2	–
29	69	M	168	68.7	1.3	25	8.6	13.5	–
30	69	M	173	66.85	1.1	24	9.8	10.7	Parkinson’s Disease
31	67	F	175	76.95	1.6	22	6.8	9.8	Peripheral neuropathy and glaucoma
32	67	F	169	82.5	1.4	21	9.4	13.8	TBI
33	72	M	172	79.95	1.1	26	9.4	10.3	–
34	76	F	160	53.5	1.3	27	9.4	13.7	–
35	72	F	167	48.85	1.2	23	7.1	10.1	–
36	67	F	158	58.2	1.1	25	10.9	13.6	–
37	71	M	185	78.4	1.3	24	10.2	17.7	–
38	66	F	164	68.75	1.3	26	9.6	11.8	–
39	74	M	189	82.75	1.0	24	8.6	14.1	–
40	76	F	154	78.1	1.2	19	7.3	10.1	–
41	52	M	176	100.65	1.2	18	10.8	11.7	TBI
42	68	F	165	72.8	1.3	25	8.4	11.1	–
43	68	M	171	84.2	1.7	25	8.2	8.75	Bilateral macular degeneration
44	66	F	165	76.25	0.9	15	13.0	18.3	MS
45	74	F	158	58.25	1.0	7	13.3	15.6	MS
46	51	M	176	93.4	1.1	21	12.0	13.8	MS
47	55	M	169	93.35	1.1	26	9.2	9.9	Unilateral vestibular hypofunction

### Physical therapist participants

**Fig. 1:**
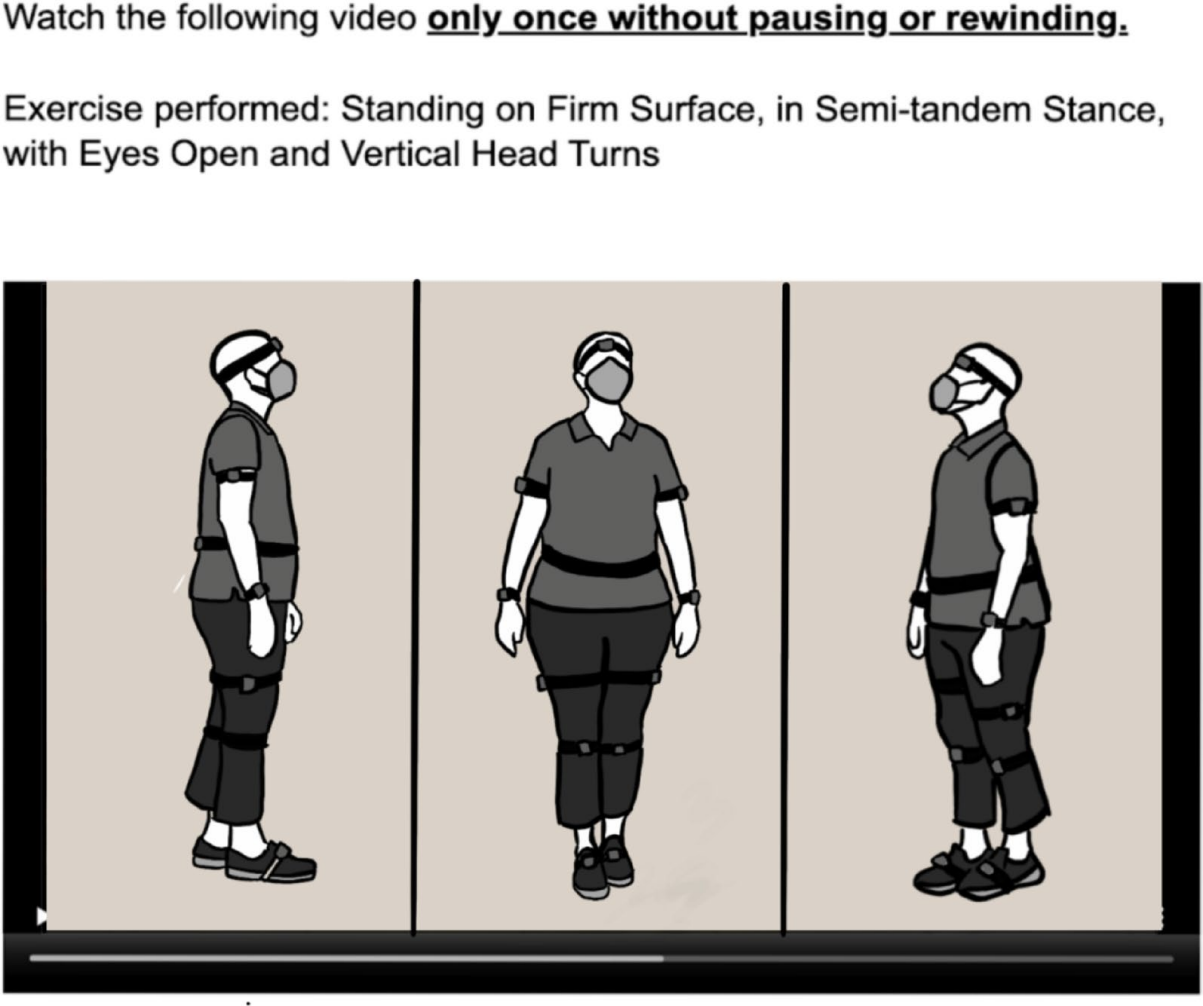
PT participants completed an online survey where they were asked to watch videos of balance exercise participants performing static standing exercises from three perspectives as illustrated in this diagram: sagittal, frontal and 45-degree view

Forty-two PTs (Table 2) were recruited to participate in a remote survey-based (Qualtrics) balance intensity assessment of the video-based recordings of the balance exercise participants. The survey prompted the PT participants to view videos of the balance exercise participants (Fig. 1) then provide ratings of balance intensity. PT participants were included if they were PTs licensed to practice in the United States, had professional experience treating individuals with balance disorders/concerns, and were able to read and comprehend English.

**Table 2:** Summary of PT participant characteristics

PT participant characteristics	
Sex	
Female	*n* = 38
Male	*n* = 4
Level of experience	
Novice (< 2 years)	*n* = 3
Intermediate (> 2 and < 12 years)	*n* = 26
Experienced (> 12 years)	*n* = 13
Average number of years working with individuals with balance deficits	10 (8) years
Populations treated	
Older adults	*n* = 40
Individuals with vestibular dysfunction	*n* = 30
Individuals with concussions or traumatic brain injury (TBI)	*n* = 32
Individuals with Parkinson’s Disease (PD)	*n* = 26
Individuals with spinal cord injury (SCI)	*n* = 10
Individuals with cerebrovascular accidents (CVA)	*n* = 35
Individuals with multiple sclerosis (MS)	*n* = 22

PT participants were classified by levels of experience into Novice (< 2 years), Intermediate (> 2 and < 12 years) and Experienced (> 12 years)

### Data collection

#### Balance training protocol

During the single balance training session balance exercise participants performed up to eight different static standing exercises with the goal of maintaining one’s balance and remaining as still as possible under various sensory conditions. Each balance exercise included a combination of the variables listed below (Table 3**)**. Balance exercises were selected by a study team member and adapted to the individual participant’s abilities to sample each participant’s balance performance at various levels of intensity informed by a conceptual framework for balance exercise progression developed by Klatt et al. [1]; no adverse events occurred. Balance exercise participants were asked to perform three 30-second repetitions of each exercise. Balance exercise participants were allowed to take breaks between repetitions as needed. All repetitions were video recorded and used for balance intensity assessment. After each repetition of an exercise, balance exercise participants were asked to provide a self-rating of their perceived balance performance on a balance self-assessment scale [35] **(**Table 4), and report whether they stepped out of their prescribed stance during the repetition. Step outs were defined as any instance when the balance exercise participant had to shift their feet to modify their stance (e.g., from tandem stance to wide support).

The balance training sessions were video-recorded to capture balance exercise participants’ performance on exercises from three perspectives (sagittal, frontal, and isometric views). These videos were then stitched and synchronized to produce short 30-second videos (Fig. 1) that were remotely reviewed by PT participants for the purpose of evaluating them. To evaluate the exercises, PT participants filled out a survey that prompted them to watch videos of balance exercise participants performing balance exercises and then provide ratings of their performance. The survey design and videos are further described under the Data Collection section “Balance evaluation protocol”.

**Table 3:** Overview of balance exercise sensory condition variables

Variable	Modifications
Surface	Firm, foam, rocker board, Bosu ball
Stance	Feet apart, feet together, semi-tandem, tandem, single leg
Eyes	Open, closed
Head turns	None, horizontal, vertical

**Table 4:** Self-assessment balance intensity scale [35]

Rating	Description
1	I feel completely steady
2	I feel a little unsteady or off-balance
3	I feel somewhat unsteady or like I may lose my balance
4	I feel very unsteady or like I will lose my balance
5	I lost my balance

#### Balance evaluation protocol

PT participants filled out a survey where they were prompted to view 30-second videos of balance exercise participants performing balance exercises. Each short video corresponded to one repetition of one exercise performed by the balance exercise participant. PT participants were asked to view each video once without pausing or rewinding, then provide a rating of the balance exercise participant's performance using a balance assessment scale [35] **(**Table 5) similar to the self-assessment scale, but using language centered around the evaluator’s as opposed to the exerciser's perspective. PT participants were randomly assigned up to five balance exercise participants to evaluate. For each balance exercise participant a PT evaluated, they were asked to view up to four exercise variations, with three repetitions of each exercise, up to 12 individual videos reviewed per balance exercise participant. The mean PT participant rating for each exercise repetition was used as the ground truth (GT) label for the automatic assessment ML-based model. Aggregate (mean) ratings of balance intensity were used as GT labels in the training and evaluation of the ML models presented in this study to capture overall clinical consensus.

**Table 5:** PT participant assessment balance intensity scale [35]

Rating	Description
1	Independent with no sway
2	Supervision with minimal sway
3	Close supervision with moderate sway
4	Requires physical assistance or positive stepping strategy after 15 s
5	Unable to maintain position with assistance or step out in the first 15 s of the exercise

### Exercise encodings

Balance exercise sensory condition variables (Table 3) such as surface, stance, eyes, and head turns were encoded following a label encoding scheme whereby each variable was assigned an integer value. For example, the variable "surface" could receive values between 1 and 4, whereby 1 corresponded to “firm” and 4 corresponded to “Bosu Ball”. Variable categories were organized in ascending order based on the expected added challenge provided by the sensory condition variable, which was informed by a balance training progression framework developed in prior work by Klatt et al. [1]. Label encoding could have introduced an implicit ordinal relationship between the categories, which was intentional for this study. By encoding exercise parameters based on their variables (i.e., surface, stance, eyes, head turns) as opposed to using unique exercise identifiers, the models trained on this dataset were able to learn trends across different exercises based on their common variables (e.g., exercises with eyes closed tend to be harder than exercises with eyes open).

#### Signal processing

Balance exercise participants wore a set of 13 wearable IMUs (Opal, APDM Inc., Portland, OR, USA) placed on the head, upper back, lower back, arms, wrists, thighs, shanks, and feet. The IMUs collected synchronized tri-axial acceleration and angular rate time-series measurements at a sampling rate of 128 Hz.

**Fig. 2:**
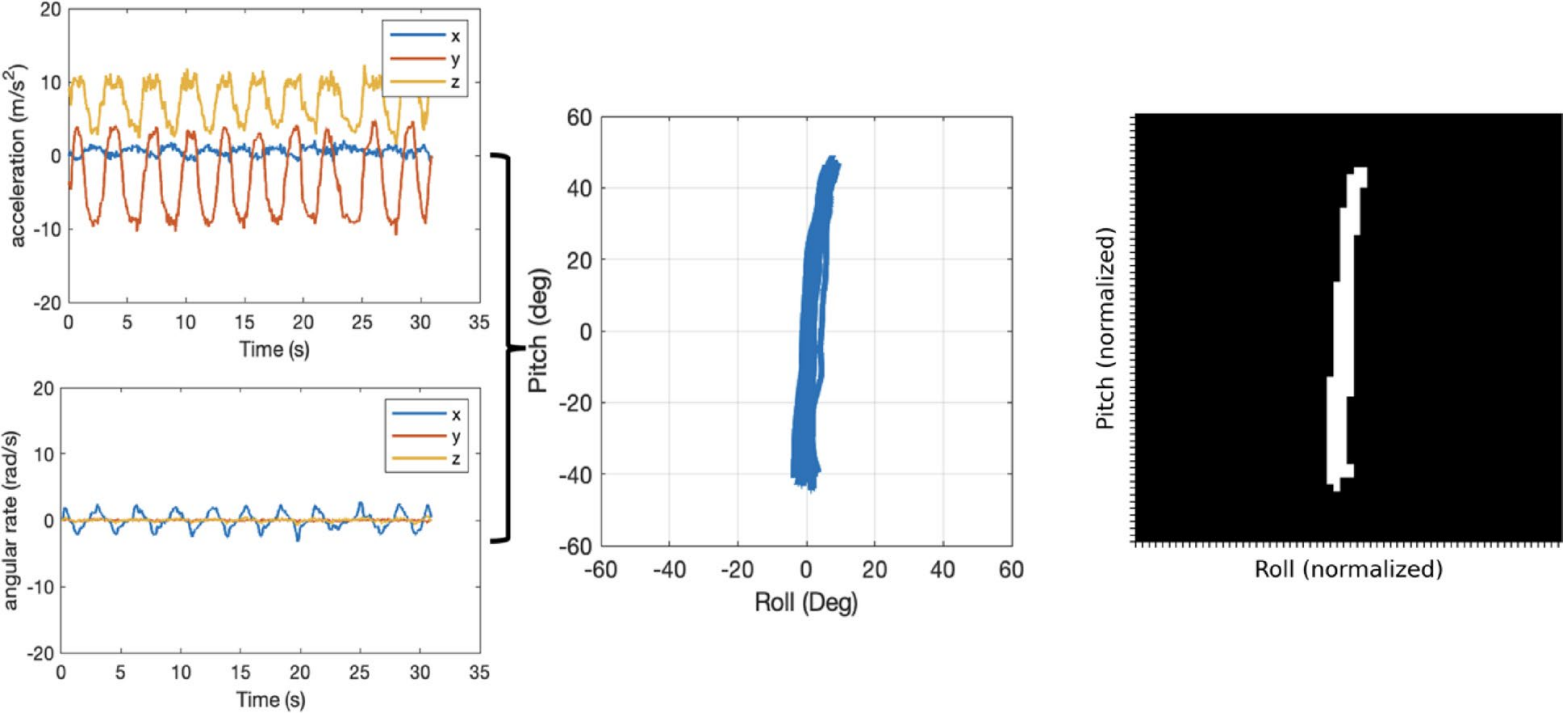
Example of IMU raw signals. In this example, data were collected from a head-mounted IMU during a 30-second exercise where an exemplary balance exercise participant stood on a foam pad with eyes open and feet together while performing vertical (pitch) head turns

Raw sensor data consisting of three-axial linear accelerations and angular velocities collected from the IMUs were clipped to 30-second samples (consistent with the prescribed duration of the exercises), each corresponding to a single repetition of a standing exercise. The raw sensor data were processed using an extended Kalman filter (EKF) and calibrated to body axes following static sensor-to-segment alignment approaches described by Nguyen et al. [40] to extract angular orientation estimates in the pitch and roll directions and produce two-dimensional (2-D) stabilogram representations of the movements of various body segments in the pitch and roll directions (Fig. 2). Similarly, 2-D stabilogram representations of the raw linear accelerations and angular velocities were also produced. The stabilogram representations of acceleration, angular velocities, and angles were used to capture postural stability in the anterior–posterior and medial–lateral directions similar to data representations found by Kamran et al. that resulted in higher ML model performance in predicting balance intensity ratings [36].

Before using these 2-D data representations as inputs to the ML models developed in this study, their values were normalized (to mean 0, std 1) based on the training dataset to avoid introducing bias in the model due to variations in participants’ ranges of motion. The 2-D stabilograms were then discretized into 64 × 64 grids, whose truncated domains encompassed − 3.2 to + 3.2 standard deviations from the mean of the training data set (99.86% of the data domain) (Fig. 2).

#### Machine learning

##### Dataset

For each repetition of an exercise, a corresponding set of inputs was created (up to 8 exercises x 3 repetitions per balance exercise participant = up to 24 repetitions per balance exercise participant). The inputs included features describing the balance exercise participants (age, sex, height, weight), the exercise variables (encodings of surface, stance, eyes, and head turns), and the 2-D stabilograms describing orientation derived from each of the 13 IMUs worn by the balance exercise participant. Finally, each repetition received a GT label (rating on the 1–5 scale) corresponding to the mean rating given by PT participants for that repetition. To address imbalances in the dataset, data weighing was applied based on the rating frequency. Imbalances due to exercise types were partially alleviated by encoding exercises into multiple parameters, such as stance, surface, stance, eyes and head turns (Table 3).

##### Model architecture

**Fig. 3:**
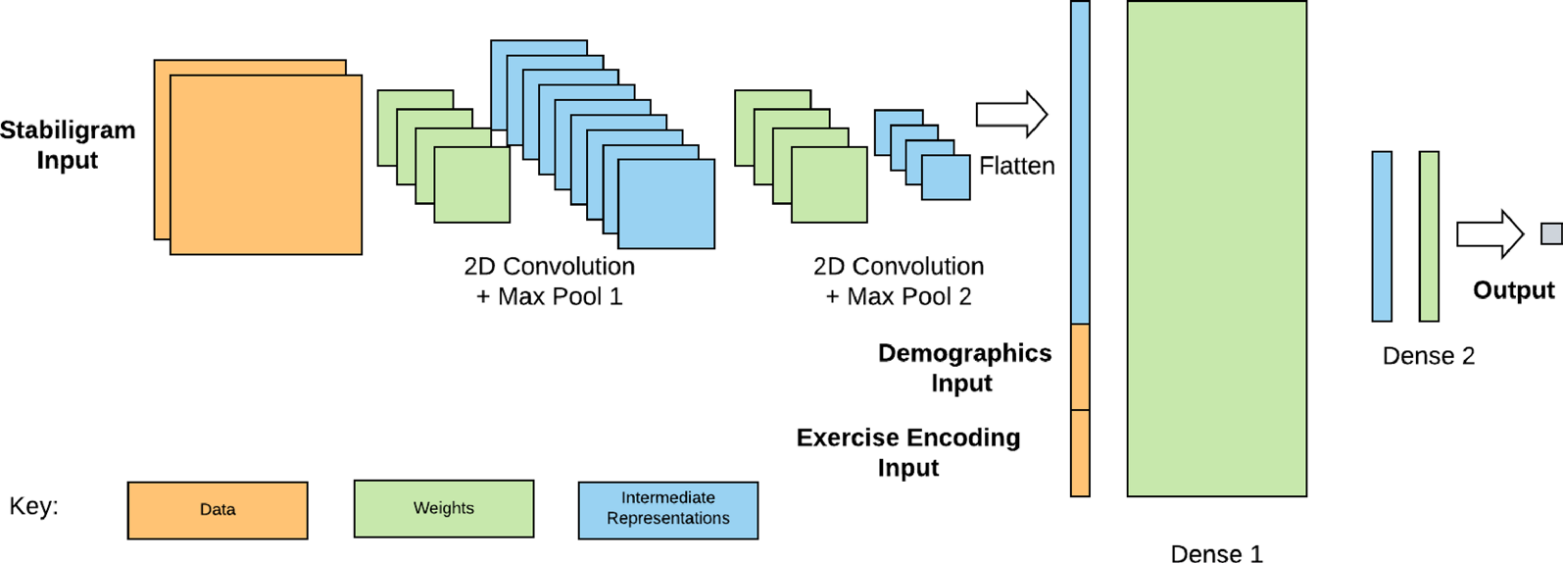
Overview of CNN-based model architecture. 2-D stabilogram representations of kinematic data [32] inputs were passed to 2-D CNN layers (two layers with the same number of neurons, each with a successive maxpool layer), and vectors with regular features describing information about the exercise variables and balance exercise participant descriptors were passed directly to the first dense layer. The two dense layers had 32 and 8 neurons, respectively

A 2-D CNN model architecture was implemented (Fig. 3) using 2-D convolutional kernels to automatically extract relevant information from 2-D inputs (e.g., 2-D stabilograms). The model used a smooth transform to output a continuous prediction with bounds (0.5, 5.5). Model hyperparameters such as the number of CNN {8, 16, 32} neurons per CNN layer, dense neurons {8, 16, 32} per dense layer, and the dropout rate {0.0, 0.2, 0.5} were selected following a hyperparameter tuning process. The hyperparameter optimization was performed using a simple grid search over the parameter ranges. The final set of hyperparameters for each model was selected by finding the setting that minimized the RMSE on the validation set, as described under the Model Training section. We chose a CNN architecture for this study due to (1) its ability to accommodate 2-D stabilograms (which are 2-D static images, not naturally handled by RNNs or transformers) that carry interpretable clinical information summarizing the participant’s movement during the entire exercise, and (2) our previous study by Kamran et al. [32] which found CNNs to yield good performance.

##### Model training

Models were trained with the typical regression loss, mean squared error (MSE), as the loss function and root mean squared error (RMSE) were monitored and reported. Both MSE and RMSE are generally accepted as the default loss functions for training and validation for models with continuous outputs (i.e., regression tasks) by the statistics and computer science communities. They bring advantages of: (1) penalizing larger errors more, (2) being differentiable, and (3) being mathematically linked to maximum likelihood. RMSE additionally yields an interpretable value, as it has the same units as the target variable. The gradient-based optimizer ADAM was used with a learning rate of 0.001.

Model training and validation were performed following a stratified five-fold cross-validation process at the level of balance exercise participants to assess model generalizability across different balance exercise participants (i.e., ability to predict ratings on new balance exercise participants that were not part of its training set). Therefore, all data from each balance exercise participant were assigned entirely to a single fold, ensuring that no data from the same balance exercise participant appeared in both training and evaluation sets. Stratification was performed to maintain a balanced distribution of rating scores across folds. In each of the five iterations of cross-validation:

- 30–31 balance exercise participants were used for training
- 7–8 were used for validation (for hyperparameter tuning)
- 9–10 were used as a held-out test set to assess generalization performance,

corresponding roughly to 20% testing, with the remainder being split 80%-20% (64%-16% overall) between training and validation, respectively. All splits were generated randomly at the balance exercise participant level, ensuring no data leakage from any given balance exercise participant across splits and simulating the real-world scenario of predicting ratings for entirely unseen balance exercise participants.

##### IMU selection process

Given the goal of identifying the best performing ML models based on the number and placement of IMU sensors, we searched for the most promising, high-performing IMU combinations through a “greedy” sequential forward selection procedure as follows, since building ML models for an exhaustive listing of all possible combinations from the 13 IMUs ($\:{2}^{13}-1$ combinations) was intractable.

1) We started by building ML models for each of the 13 single-IMU cases (i.e., each ML model used data from only one of the 13 IMUs).

2) Next, we expanded to two-IMU models by first identifying the three highest performing single-IMU models (based on validation RMSE averaged over all validation folds), and then augmented each of these cases with data from one of the remaining possible IMUs.

3) The same expansion process was repeated to expand to models using three and more IMUs. To ensure that each sensor combination was given an opportunity to perform as well as possible, model training and hyperparameter tuning was repeated for each new sensor combination.

##### Model evaluation

**Fig. 4:**
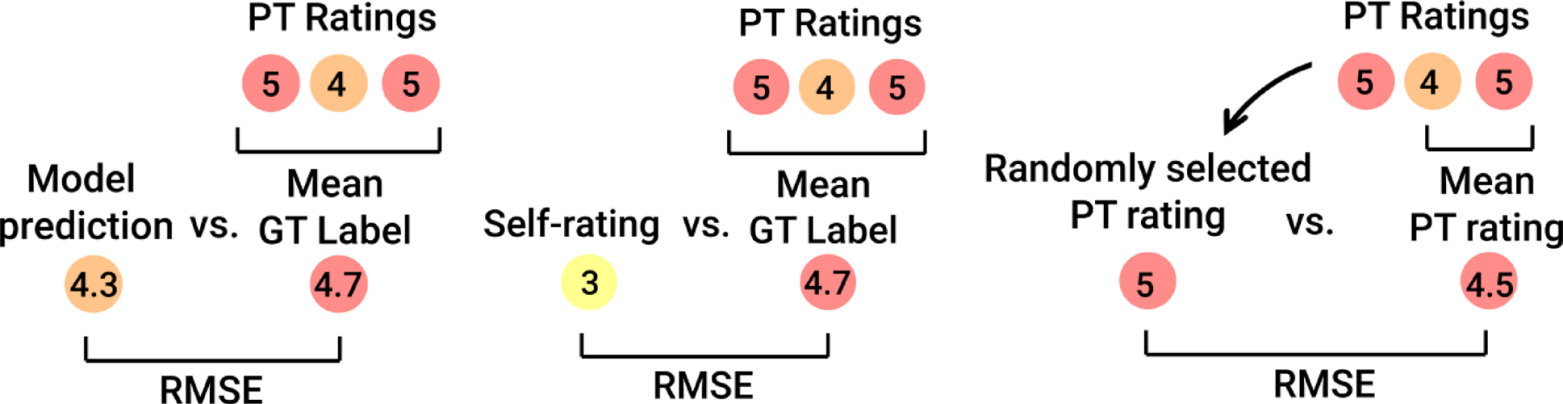
Summary of model evaluation metrics. Model performance was assessed by calculating RMSE values of model predictions relative to GT labels and compared to benchmarks representing self-rating and PT RMSEs

Models were evaluated using “Model prediction RMSE” (left diagram in Fig. 4). Multi-class accuracies and F-1 scores were also reported by rounding the continuous model predictions to the nearest integer {1, 2, 3, 4, 5} to form a classification prediction metric from the models. Continuous model outputs were rounded to the nearest integer solely for the purpose of computing standard categorical metrics such as accuracy and F-1 score. We recognize that these metrics, as well as others like sensitivity and specificity, treat all misclassifications equally (e.g., being off by 1 is penalized the same as being off by 4), which is not ideal for ordinal or cardinal outcomes. In our context, where labels are discrete but ordered, standard classification metrics cannot capture the degree of prediction error. However, accuracy was included due to its ease of interpretation for a broad audience, and F-1 was used for its ability to balance precision and recall, particularly under class imbalance.

GT label ratings were defined by selecting the mean rating among all ratings provided by PT participants for a given data point (i.e., a particular balance exercise participant, exercise, and repetition set). Each repetition in the dataset was reviewed by at least one PT participant and up to five PT participants. PT participant assessments were considered as the GT label as they reflected assessments that were clinically informed, and consistent with established standards of care. While self-reported ratings provided valuable insight into the balance exercise participants’ perceptions, they can be influenced by factors such as confidence, emotional state, or lack of awareness of movement quality, all of which may introduce substantial bias or variability. In contrast, PTs are trained to observe and evaluate movement patterns, posture, and balance using standardized clinical criteria, making their assessments more reliable indicators of actual performance. Our goal was to train a model that reflects clinical judgment and could support home-based training where direct PT assessment may not be accessible. Therefore, we prioritized PT participant assessments as the GT to ensure that the model learned to approximate expert evaluations rather than subjective balance exercise participant evaluations.

Additional benchmark RMSE values were calculated using bootstrapping techniques to contextualize model performance: (1) “Self-ratings RMSE” (middle diagram of Fig. 4) was estimated by comparing balance exercise participant self-ratings against the model GT labels, and (2) “Random PT RMSE” (right diagram of Fig. 4) was estimated by randomly selecting one PT participant's rating as the predicted label for each data point, and then calculating the RMSE of those predictions relative to the mean PT participant ratings but with the selected PT participant rating removed (similar to the approach in [37]) in order to avoid any leakage of information from the GT to the prediction; the random PT participant selection was then repeated 1000 times and results averaged.

Finally, we used a paired two-tailed t-test with a significance level of 0.05 to compare Model predictions RMSE with Random PT ratings RMSE values, as well as Self-rating RMSE with Random PT ratings RMSE values.

## Results

### PT participant ratings and self-ratings distributions

**Fig. 5:**
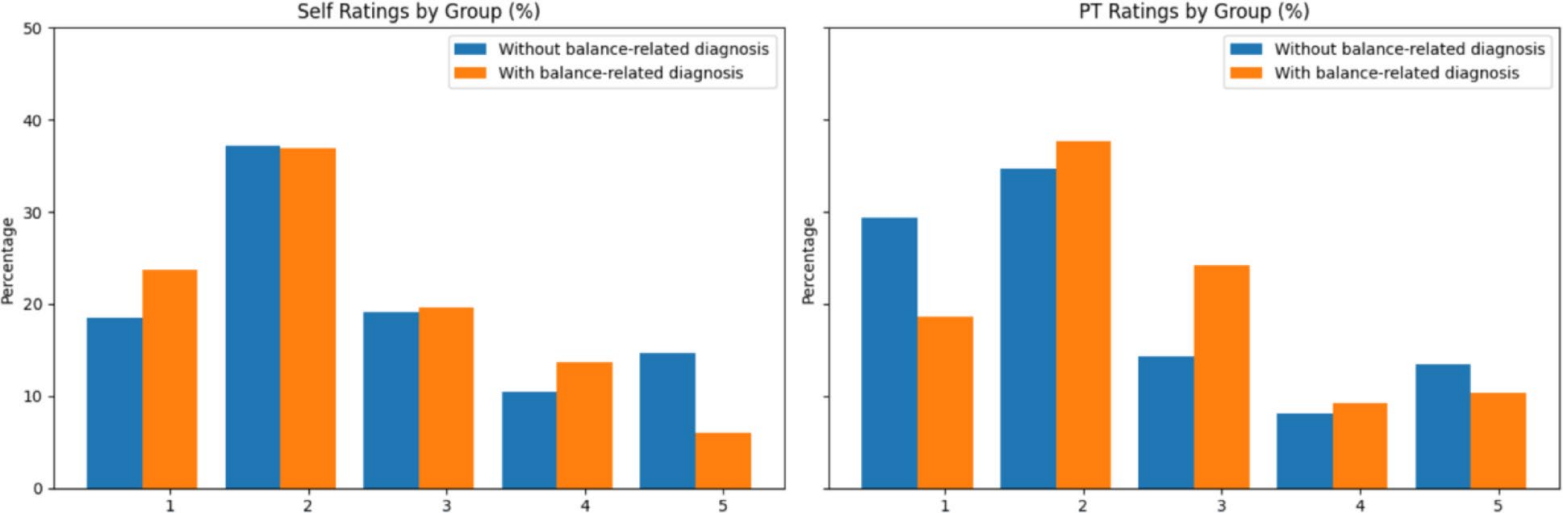
PT participant and self-rating histograms. Both PT participants and balance exercise participants reported balance intensity ratings across all levels on the rating scale but had higher numbers of ratings of lower intensity (1–2) than other levels of intensity

The dataset collected in this study included 1,494 PT participant ratings and 888 self-ratings. The distribution of these ratings across levels on the balance intensity scale is shown in Fig. 5. The distributions were weighted towards lower intensity ratings with a mode rating of 2.

### Model performance based on using all IMU placements

**Fig. 6:**
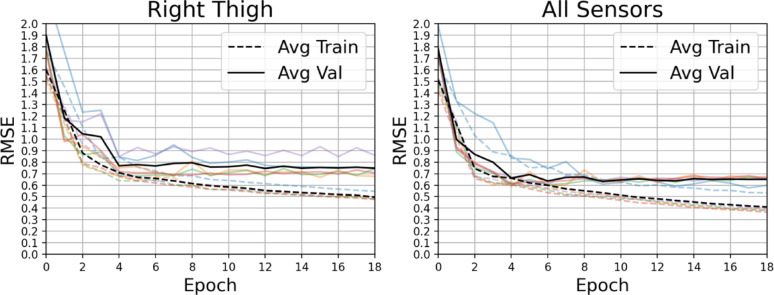
Training and validation curves shown at each training epoch. Lighter, colored curves show metrics for each cross validated model while black curves show the training and validation metrics averaged across all cross-validation folds. A separation appears at later epochs, consistent with overfitting to a small training set

Model predictions RMSE (for model trained on data from all 13 IMUs), dummy regressor RMSE, Self-rating RMSE, and Random PT ratings RMSE are reported in Table 6. The dummy regressor was based on naively predicting p(Y) rather than p(Y|X); in other words drawing from the distribution of labels without respect to input features. The dummy regressor serves to demonstrate, particularly in class-imbalanced data, that the regression task is not trivial and that intelligent use of input features is warranted. Model predictions as well as self ratings and PT ratings all showed substantial improvement over the dummy regressor. Models trained on kinematic data from the full set of 13 IMUs achieved a mean RMSE of 0.66 [0.62, 0.69] (Table 6). Training curves for the best single sensor and the set of all sensors are shown in Fig. 6. These models achieved lower RMSE compared to both balance exercise participant self-ratings and individual ‘Random PT’ ratings when evaluated against the GT (mean PT participant ratings). This finding indicates that the model’s predictions were, on average, more aligned with the consensus PT participants' judgment than either balance exercise participant self-assessments or the rating of a single PT participant. While both balance exercise participant self-ratings and individual PT participant ratings differed from the GT, the model’s performance suggests it may reduce individual variability and approximate the aggregate clinical perspective more closely than a single human rater or PT participant.

**Table 6:** Model predictions RMSE, Self-ratings RMSE, and Random PT ratings RMSE

	Model predictions	Dummy regressor predictions	Self-ratings	Random PT ratings
Mean RMSE [95% CI] (↓ lower is better)	0.66* [0.62, 0.69]	1.80* [1.75, 1.84]	0.93* [0.88, 0.99]	0.74 [0.72, 0.76]

The ML model used a CNN architecture and was trained on data from all 13 IMUs. The Model prediction RMSE was lower than Self-ratings RMSE and Random PT ratings RMSE. (*) indicates statistically significant differences relative to Random PT RMSE values (*p* < 0.05)

**Fig. 7:**
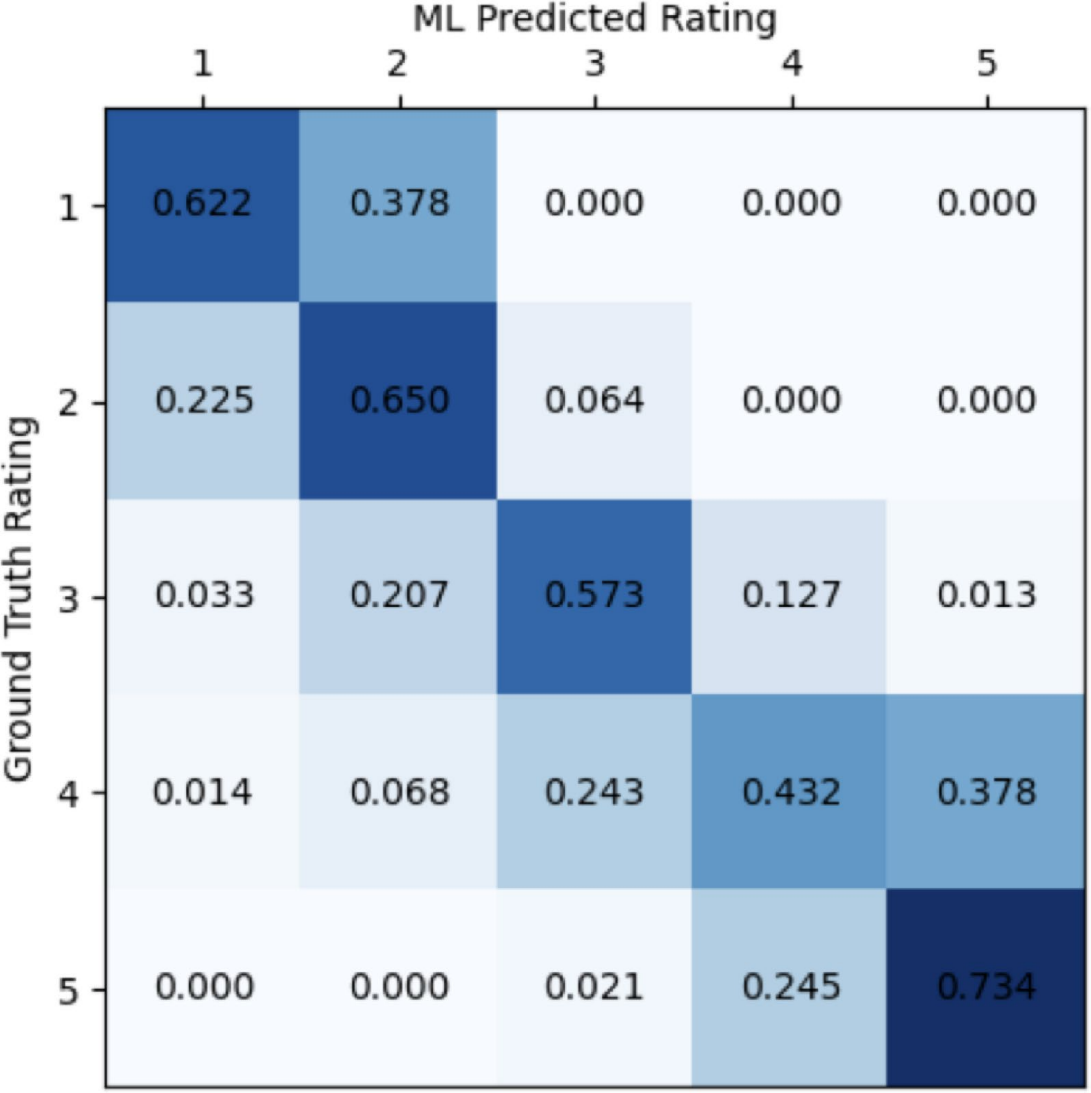
Confusion matrix associated with classification predictions made from the ML model using all IMU placements

The model predictions were translated into classification predictions by rounding the results to the nearest 1–5 integers. Classification performance in terms of F-1 scores, accuracy, and confusion matrix for the models trained on all 13 IMU placements were reported in Table 7. The models achieved a mean accuracy of 64%. Figure 7  shows the full confusion matrix. Model predictions fell within one point of the GT rating for all samples when the GT rating was 1 or 2. Only a few (< 10) predictions fell more than one point away from the GT rating when the GT rating was 3, 4 or 5.

### Effect of number and placement of IMUs on model performance

Test RMSE values for models trained on each of the 13 individual IMU placements are reported in Table 7. Mean RMSE values varied between 0.68 and 0.90 depending on the IMU placement. Results from the IMU down-selection process are shown in Fig. 7. As the number of IMUs included in the models increased beyond four sensors, only marginal gains in RMSE values were achieved. The best validation performance (lowest RMSE) for one sensor was achieved with an IMU placed on the right thigh. The best three-sensor model performance corresponded to models trained on data from IMU placements on the left thigh, right thigh and lumbar, which had RMSE values lower than those observed with the Random PT rating RMSE reported in Table 6.

**Table 7:** Summary of model performance results in terms of test RMSE, F-1 and accuracy for ML models trained on kinematic data from single-IMU placements as well as for the full 13-IMU configuration (i.e., all sensors)

Sensor	RMSE (↓) Mean [95% CI]	F-1 (↑) Mean [95% CI]	ACC (↑) Mean [95% CI]
Head	0.82 [0.77, 0.87]	0.31 [0.11, 0.66]	0.47 [0.43, 0.50]
Left Arm	0.77 [0.74, 0.80]	0.41 [0.16, 0.69]	0.53 [0.50, 0.56]
Left Foot	0.78 [0.74, 0.82]	0.31 [0.18, 0.60]	0.43 [0.40, 0.47]
Left Shank	0.78 [0.75, 0.82]	0.45 [0.23, 0.65]	0.51 [0.47, 0.54]
Left Thigh	0.77 [0.71, 0.82]	0.45 [0.12, 0.65]	0.43 [0.40, 0.46]
Lumbar	0.80 [0.75, 0.85]	0.38 [0.21, 0.62]	0.47 [0.43, 0.50]
Left Wrist	0.80 [0.77, 0.84]	0.44 [0.32, 0.66]	0.50 [0.47, 0.54]
Right Arm	0.79 [0.76, 0.82]	0.43 [0.31, 0.66]	0.52 [0.49, 0.56]
Right Foot	0.80 [0.76, 0.84]	**0.48 [0.30**,** 0.74]**	**0.58 [0.54**,** 0.61]**
Right Shank	0.81 [0.76, 0.84]	0.42 [0.26, 0.59]	0.48 [0.45, 0.51]
Right Thigh	**0.76 [0.73**,** 0.80]**	0.47 [0.33, 0.70]	0.53 [0.50, 0.56]
Right Wrist	0.79 [0.74, 0.84]	0.37 [0.25, 0.60]	0.45 [0.42, 0.48]
Upper Back	0.79 [0.76, 0.82]	0.42 [0.29, 0.69]	0.50 [0.47, 0.53]
**All Sensors**	**0.66 [0.62**,** 0.69]**	**0.61 [0.37**,** 0.74]**	**0.64 [0.61**,** 0.66]**

## Discussion

**Fig. 8:**
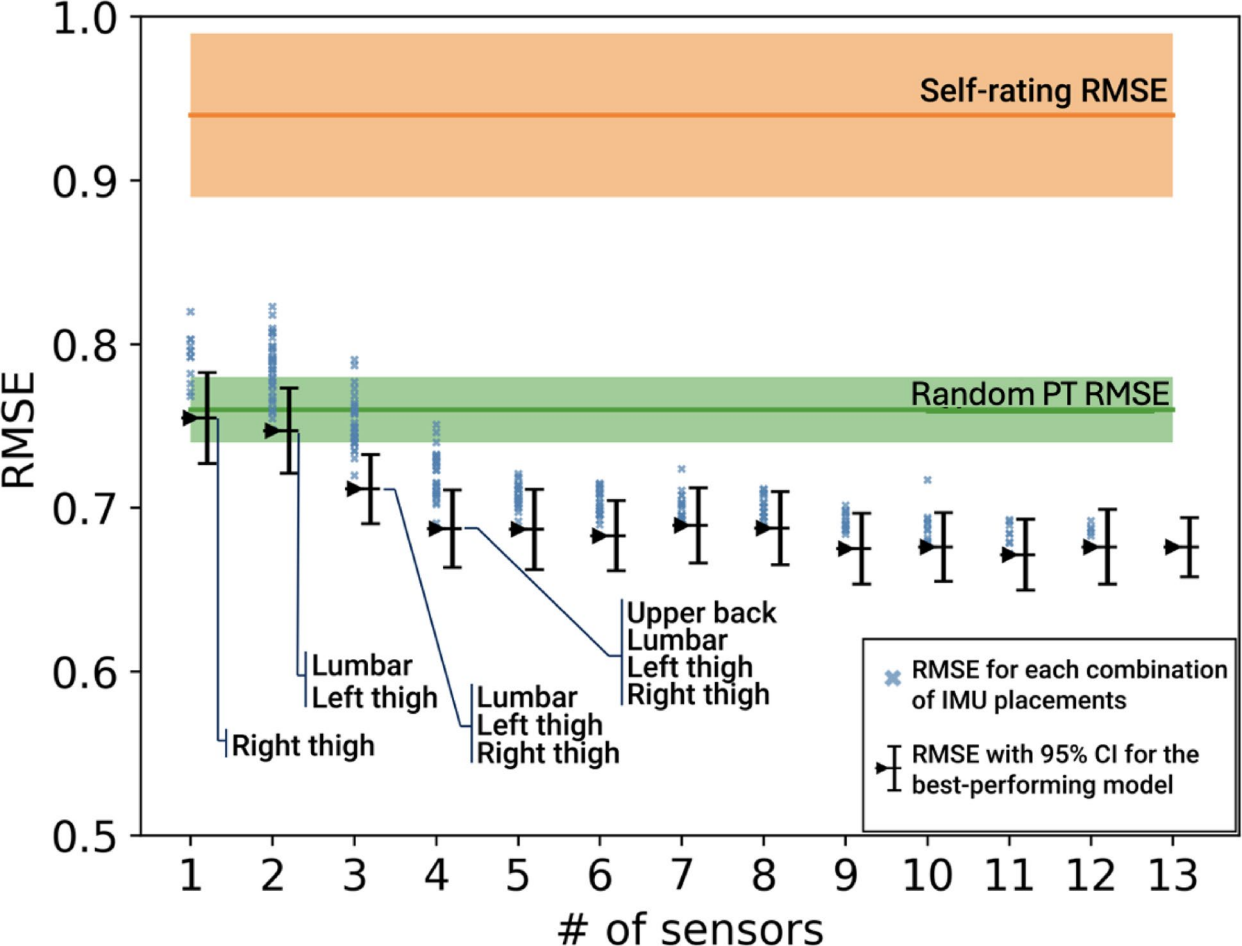
Validation RMSE values by the number of IMUs used in training CNN models. Each black point represents the best combination of IMU placements for that number of IMUs, with error bars showing the 95% CI. Blue points show the mean performance for each suboptimal model for that number of IMUs. The orange band shows self-rating performance, and the green band shows the Random PT performance, each with 95% CIs

In this study, we trained ML models with a CNN architecture to automatically predict PT participant ratings of balance intensity on a 1–5 scale using IMU-derived orientation angles. CNN models trained on data from all 13 IMU placements were able to predict balance intensity ratings with a mean test RMSE of 0.66 [0.62, 0.69] and an accuracy of 64% [61%, 66%]. The model test RMSE was significantly lower than the RMSE estimate for the Radom PT based on expected inter-rater variabilities (RMSE = 0.74 [0.72, 0.76]) (Table 6). While RMSE does not directly quantify inter-rater agreement or reliability, this comparison provides a practical reference point, suggesting that the model’s prediction error falls within the range of disagreement typically observed between individual PT participants and the group consensus. Future research could explore complementary methods such as Bland-Altman analysis to better characterize agreement and potential bias among model predictions and clinician ratings, extending beyond RMSE-based comparisons to deepen understanding of model-clinician alignment. By contrast, the RMSE values associated with self-ratings of balance intensity (RMSE = 0.93 [0.88, 0.99]) were significantly higher than those observed for individual PT participant ratings relative to the consensus (Random PT RMSE = 0.74 [0.72, 0.76]) (Table 6), suggesting that self-ratings were less accurate in approximating the consensus PT participant rating, a trend that has also been reported in previous studies [34–36]. Together, these findings suggest that the predictions of balance intensity ratings produced by the presented models can provide accurate estimates of intensity during static standing exercises (Fig. 8).

While our findings indicate that self-ratings deviate more from the PT consensus than either model predictions or Random PT ratings, this does not imply that self-assessments are invalid or unimportant, particularly in the context of patient-centered rehabilitation. Self-perceived balance intensity may reflect internal factors such as effort, confidence, or fear, which are not directly observable by clinicians or measurable by sensors. These subjective insights can be clinically meaningful and may complement externally rated assessments. Future work should explore how self-assessments and PT ratings diverge and interact, and whether models can be developed to support both perspectives in adaptive, patient-centered feedback systems.

Model performance for the CNN models developed in this study based on kinematic data from all 13 IMU placements achieved a comparable accuracy (ACC = 64% [61, 66]) to values reported by Bao et al. (ACC = 64% +/- 11%) on the same 1–5 balance intensity scale. The number and placement of IMUs influenced model performance, with diminishing returns as additional IMUs were added beyond four (Fig. 8). This finding indicated that while additional sensors were beneficial in improving predictive performance, a single IMU placed on the right thigh achieved RMSE values comparable to random PT RMSE values, and a set of three IMUs including both thighs and the lower back (lumbar) was sufficient to achieve an RMSE below the threshold defined by random PT RMSE values. Furthermore, several single IMU placements (Table 7) including the left wrist (RMSE = 0.74 [0.71, 0.78]), were able to achieve RMSE values comparable to random PT RMSE values. IMU placement had an effect on model performance as it enabled models to learn from different features and compensatory reactions that occured at different body segments, in alignment with findings from prior studies [42, 43]. Beyond model performance or accuracy, the choice of number and placement of IMUs involves practical considerations as fewer and easier-to-place sensors are generally preferred [44]. While a left wrist placement did not result in the lowest single-IMU RMSE performance achieved in this study, such a placement may present advantages with respect to usability with minimal losses to accuracy as IMUs are already widely embedded in wrist-based smart devices. Prior studies [38–40] have mainly focused on lower back IMU placements to capture balance performance on static standing exercises, but findings from our study showed that additional IMU placements on the lower limbs, and specifically on the thighs enabled improved predictive performance as they captured additional markers of balance intensity [11, 45].

Limitations to this work included balance intensity evaluations based on a limited number of PT observations, subject to inter-rater variabilities. Relying on observational assessments by PT participants to evaluate balance intensity was aligned with current clinical standards of care and prior research [11, 34–36] in the absence of an objective measure of balance intensity [3]. To address this limitation, we chose to select the mean PT participant rating as a consensus rating for all PT participants who observed the same repetition and performed bootstrapping analyses to contextualize model performance with respect to inter-rater differences in the dataset (Table 6). A second limitation was the skew in the distribution of balance intensity ratings (Fig. 5). More instances of low-intensity ratings (1–2) were present in the dataset relative to higher-intensity ratings (4–5). While the dataset used in model training was re-weighted, this skew in distribution may have affected model accuracies on the less-represented rating levels (Table 7). Further development of automatic balance intensity assessment systems may be able to address this limitation by focusing on populations with known balance deficits who may be more likely to experience higher levels of balance intensity during training.

Balance intensity is a key factor in defining and customizing the dosage of balance training exercises included in rehabilitation programs. Enabling the automatic assessment of balance intensity through wearable IMUs provides an opportunity to supervise home-based training and provide feedback to exercisers in the absence of PT observations, ensuring the effectiveness of each session.

## Conclusion

IMUs can be used to monitor the intensity dosage of balance training exercises. The current study presented ML models that leveraged IMU-derived data to automatically evaluate balance intensity on 1–5 scale during static standing balance training exercises with reliable performance (RMSE = 0.66 [0.62, 0.69]). The analyses presented in this study also examined the effects of the number and placement of IMUs on predictive performance and found that one to four IMUs were sufficient to achieve good performance. The models presented in this study could be applied to support the monitoring and personalization of home-based balance training.

## Data Availability

The datasets used and/or analyzed during the current study are available from the corresponding author on reasonable request.

## Abbreviations

CI: Confidence interval
CNN: Convolutional Neural Networks
GT: Ground truth
IMU: Inertial Measurement Unit
ML: Machine learning
PT: Physical therapist
RMSE: Root-mean-square error
SVM: Support Vector Machines
